# Randomized multicenter phase III study of a modified docetaxel and cisplatin plus fluorouracil regimen compared with cisplatin and fluorouracil as first-line therapy for advanced or locally recurrent gastric cancer

**DOI:** 10.1007/s10120-015-0457-4

**Published:** 2015-01-21

**Authors:** Jinwan Wang, Ruihua Xu, Jian Li, Yuxian Bai, Tianshu Liu, Shunchang Jiao, Guanghai Dai, Jianming Xu, Yunpeng Liu, Nanfeng Fan, Yongqian Shu, Yi Ba, Dong Ma, Shukui Qin, Leizhen Zheng, Weichang Chen, Lin Shen

**Affiliations:** 1 Cancer Institute and Hospital, Chinese Academy of Medical Sciences, Beijing, China; 2 Sun Yat-Sen University Cancer Center, Guangzhou, China; 3 Department of Gastrointestinal Oncology, Key Laboratory of Carcinogenesis and Translational Research (Ministry of Education), Peking University Cancer Hospital and Institute, No 52 Fu Cheng Road, Haidian District, Beijing, 100142 China; 4 Harbin Medical University Cancer Hospital, Harbin, China; 5 Fudan University Affiliated Zhong Shan Hospital, Shanghai, China; 6 Chinese PLA General Hospital, Beijing, China; 7 307 Hospital of PLA, Beijing, China; 8 The First Hospital of China Medical University, Shenyang, China; 9 Fujian Provincial Cancer Hospital, Fuzhou, China; 10 Jiangsu Provincial Hospital, Nanjing, China; 11 Tianjin Medical University Cancer Institute and Hospital, Tianjin, China; 12 Guangdong General Hospital, Guangzhou, China; 13 PLA Cancer Center of Bayi Hospital, Nanjing, China; 14 Xin Hua Hospital Affiliated to Shanghai Jiao Tong University School of Medicine, Shanghai, China; 15 Suzhou University Affiliated First Hospital, Suzhou, China

**Keywords:** Advanced gastric cancer, Modified docetaxel and cisplatin plus fluorouracil regimen, Progression-free survival, Overall survival, Safety

## Abstract

**Background:**

The V325 study showed that docetaxel, cisplatin, and fluorouracil (DCF) prolonged overall survival (OS) of patients with advanced gastric cancer, but with a high incidence of dose-limiting toxicities. We investigated the efficacy and safety of a modified DCF (mDCF) regimen for Chinese patients with advanced gastric cancer.

**Methods:**

Untreated advanced gastric cancer patients randomly received docetaxel and cisplatin at 60 mg/m^2^ (day 1) followed by fluorouracil at 600 mg/m^2^/day (days 1–5; mDCF regimen) or cisplatin at 75 mg/m^2^ (day 1) followed by fluorouracil at 600 mg/m^2^/day (days 1–5; CF) every 3 weeks. The primary end point was progression-free survival (PFS). The secondary end points were OS, overall response rate (ORR), time-to-treatment failure (TTF), and safety.

**Results:**

In total, 243 patients were randomized to treatment (mDCF regimen 121; CF 122). Compared with CF, the mDCF regimen significantly improved PFS and OS: the median PFS was 7.2 and 4.9 months, respectively [hazard ratio (HR) 0.58, log-rank *P* = 0.0008], and the median OS was 10.2 and 8.5 months, respectively (HR = 0.71, *P* = 0.0319). Additionally, the mDCF regimen improved the parameters used as secondary objectives: the ORR was 48.7 % with the mDCF regimen versus 33.9 % with CF (*P* = 0.0244); the median TTF was 3.4 months with the mDCF regimen and 2.4 months with CF (HR = 0.67, *P* = 0.0027). Grade 3 and grade 4 treatment-related adverse events occurred in 77.3 % of patients who received the mDCF regimen versus 46.1 % of patients who received CF (*P* < 0.001).

**Conclusions:**

The mDCF regimen, compared with CF, significantly prolonged PFS and OS and enhanced ORR of Chinese patients with advanced gastric cancer. The mDCF regimen achieved efficacy comparable to that of DCF but with fewer toxicities, which is appropriate for the Chinese population.

**Electronic supplementary material:**

The online version of this article (doi:10.1007/s10120-015-0457-4) contains supplementary material, which is available to authorized users.

## Introduction

Gastric cancer is the fourth most frequent malignancy and the second leading cause of cancer-related mortality. Since the early stages of the disease are often asymptomatic, it is frequently diagnosed at an advanced stage [1, 2].

In the treatment of advanced gastric cancer, no chemotherapy combination has been accepted as the gold standard [1]. Regimens based on 5-fluorouracil (5-FU) and/or cisplatin have been shown to improve survival only modestly in patients in whom advanced gastric cancer is diagnosed [3, 4]. Furthermore, there are few high-quality clinical studies and no internationally accepted standard treatment regimen for patients with gastric cancer in China, which accounts for the 47 % of global new gastric cancer cases [1, 2].

Docetaxel has demonstrated activity against gastric cancer both as monotherapy and as a combination with other chemotherapeutic agents, especially when given together with cisplatin and 5-FU [5–7]. The phase II/III V325 study showed that the addition of docetaxel to a cisplatin and 5-FU (CF) regimen significantly improved the time to progression and the overall survival (OS) in untreated advanced gastric cancer patients [5]. However, all patients receiving docetaxel, cisplatin, and 5-FU (DCF; docetaxel at 75 mg/m^2^ and cisplatin at 75 mg/m^2^ plus 5-FU at 750 mg/m^2^) experienced at least one treatment-emergent adverse event. Despite enrollment of a young population (median age 55 years) with a good functional status (Eastern Cooperative Oncology Group performance status of 0–1), treatment-related grade 3 or grade 4 treatment-emergent adverse events occurred in 69 % of patients. Grade 3–4 neutropenia, febrile neutropenia, and diarrhea were seen in 82, 29, and 19 % of patients, respectively [8]. Similar high rates of grade 3 and grade 4 neutropenia and gastrointestinal adverse events were reported in other clinical trials using the same regimen [7]. As a consequence, the toxicity profile of the DCF regimen has limited the use of this combination.

To achieve the same efficacy with fewer adverse effects, several studies testing various modified DCF (mDCF) regimens have been conducted [9–18]. In a retrospective study that enrolled 54 patients with advanced gastric cancer, an mDCF regimen consisting of lower doses of DCF (docetaxel at 60 mg/m^2^, cisplatin at 60 mg/m^2^, and 5-FU at 600 mg/m^2^) showed efficacy similar to that of the DCF regimen [OS and progression-free survival (PFS) were 10.7 and 6.8 months, respectively] [19]. The incidence of grade 3 and grage 4 toxicities was lower than with the DCF regimen, and grade 3–4 neutropenia was reported in only 4 % of the patients. In a study performed in 107 patients with locally advanced or metastatic gastric cancer, the same mDCF regimen was compared with DCF and was also associated with a lower incidence of grade 3–4 toxicities (grade 3–4 neutropenia reported in 48.3 % of the patients in the DCF arm vs 13.6 % of the patients in the mDCF regimen arm) while maintaining a similar activity (PFS of 9.9 months vs 8.6 months; OS of 7.4 months vs 6.5 months; *P* > 0.05) [14].

However, there is still lack of randomized controlled studies on mDCF regimens in patients with advanced gastric cancer, especially in China. Therefore, on the basis of the above-mentioned studies that showed a lower incidence of toxicities for an mDCF regimen consisting of docetaxel at 60 mg/m^2^, cisplatin at 60 mg/m^2^, and 5-FU at 600 mg/m^2^, we designed a clinical trial to investigate the efficacy and safety of an mDCF regimen in chemotherapy-naïve advanced gastric cancer patients in China.

## Patients and methods

### Study design and treatment

This study was a multicenter, prospective, randomized, open-label, phase III trial (NCT00811447). It was conducted to compare the efficacy and safety of the dosage-modified DCF regimen versus CF regimes in patients with advanced gastric cancer or gastroesophageal junction cancer.

Random assignment was centralized and stratified for center, liver metastases, prior gastrectomy, Karnofsky performance status (KPS) (80 or above vs below 80), and weight loss during the previous 3 months (5 % or less vs more than 5 %). Eligible patients were randomly assigned (1:1) to receive either docetaxel (Taxotere; Sanofi-Aventis, Paris, France) at 60 mg/m^2^ (1-h intravenous infusion) plus cisplatin at 60 mg/m^2^ (1- to 3-h intravenous infusion) on day 1, followed by 5-FU at 600 mg/m^2^/day (continuous intravenous infusion) for 5 days (mDCF regimen) or cisplatin at 75 mg/m^2^ on day 1 followed by 5-FU at 600 mg/m^2^/day for 5 days (CF regimen). Treatment was given in 3-week cycles. During the study, the dose modification criteria were predefined and were based on toxicities. All patients received appropriate hydration and patients in the mDCF regimen arm also received corticosteroids as premedication. Treatment continued until there was disease progression, unacceptable toxicity, death, or consent withdrawal.

The study was approved by the Institutional Review Board of each participating center or the competent authority and the Ethics Committee. The study was conducted in full accordance with the International Conference on Harmonization Good Clinical Practice guidelines and the Declaration of Helsinki. All patients provided written informed consent before any study procedure.

### Patient population

Major inclusion criteria were age 18 years or older; histologically proven gastric or gastroesophageal junction adenocarcinoma; measurable and/or assessable metastatic disease according to Response Evaluation Criteria In Solid Tumors (RECIST) version 1.0 [20]; KPS higher than 70; no prior palliative chemotherapy; 6 weeks or longer following radiotherapy and 3 weeks or longer following surgical intervention; and adequate hepatic, renal, and hematologic function. The major exclusion criteria were other concomitant cancer, neuropathy, brain or leptomeningeal involvement, uncontrolled significant comorbid conditions, or if the patient could not comprehend the purpose of the study and could not comply with its requirements.

### Study assessment

Assessments were performed at the time of the enrollment, and every 6 weeks after the administration of the study drugs. Tumor response was defined as complete response (CR), partial response (PR), stable disease (SD), and progressive disease (PD) according to RECIST and was assessed by the investigators. PFS was measured from the day of the random assignment to the first evidence of progression or death. Survival was defined as the time from the date of random assignment to the date of death from any cause. The tumor response rate (RR) was calculated per treatment arm as the proportion of randomized patients having a confirmed best response of PR or CR. Time-to-treatment failure (TTF) was defined as the time from the date of randomization to the date of failure (progression, death, or any other causes of treatment discontinuation). Toxicities were evaluated weekly and were graded according to the National Cancer Institute of Canada Common Toxicity Criteria (NCIC-CTC) version 3.0 [21].

### Statistical analysis

The primary objective was to demonstrate superiority of DCF compared to CF in terms of PFS using a stratified log-rank test with a two-sided 5 % significance level. We considered that the superiority would have been proven if the mDCF regimen prolonged the PFS by at least 1.9 months compared with CF. This calculation was based on the results of the V325 study, which showed a PFS of 3.7 months with CF and 5.6 months with DCF [5]. We estimated that at least 187 events should be observed in 200 patients to have an 80 % power to reject the null hypothesis. Assuming a loss to follow-up of 15 %, we calculated that 240 subjects (120 subjects per treatment arm) were required. The study duration was 30 months, and subject accrual occurred in the first 18 months. The analysis of the primary end point was performed in the intention-to-treat population.

The major secondary end points included OS, overall RR (ORR), TTF, and safety. The Kaplan–Meier curve was used to describe survival data. PFS and OS were compared between arms using the stratified log-rank test as well as the Cox proportional hazards model. ORRs were compared using Fisher’s exact test. Safety analyses were based on the safety sets defined as all patients who received at least one dose of the study medication and had at least one follow-up safety assessment. Safety analyses included all adverse events, as well as the events possibly or probably related to study medication, and were performed using Fisher’s exact test.

For the stratified log-rank test, the parameters used for the stratification of the randomization were included as covariates in the model: liver metastasis (yes, no), prior gastrectomy (yes, no), weight loss in the past 3 months (5 % or less, more than 5 %) and KPS (80 or above, below 80).

## Results

### Patient demographic and baseline characteristics

**Fig. 1:**
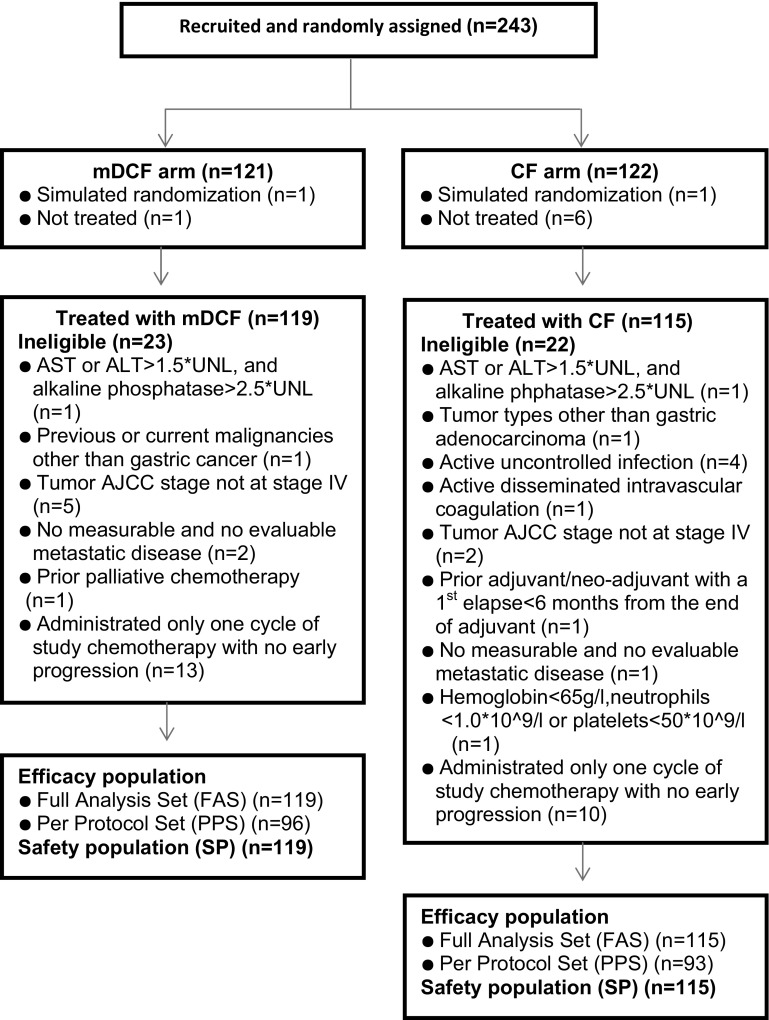
The Consolidated Standards of Reporting Trials (CONSORT) diagram depicting the trajectory of the trial. *AJCC* American Joint Committee on Cancer, *ALT* alanine transaminase, *AST* aspartate transaminase, *CF* cisplatin and fluorouracil, *mDCF* modified docetaxel, cisplatin, and 5-fluorouracil regimen, *UNL* upper normal limit

A total of 243 patients from 15 centers across China were eligible and were randomized to treatment (mDCF regimen 121; CF 122); 234 patients received the allocated combination regimen (mDCF regimen 119; CF 115) and were included in the full-analysis population. The study flow chart is shown in Fig. 1. Patients in both arms were comparable in terms of demographic and baseline characteristics (Table 1).

**Table 1:** Demographic and baseline characteristics of study participants (*n* = 234)

Characteristics	DCF (*n* = 119)	CF (*n* = 115)	Total (*n* = 234)
Male sex	81 (68.1 %)	88 (76.5 %)	169 (72.2 %)
Age			
Median (years)^a^	56.6 (19–80)	55.5 (33–74)	56.1 (19–80)
<65 years	99 (83.2 %)	96 (83.5 %)	195 (83.3 %)
≥65 years	20 (16.8 %)	19 (16.5 %)	39 (16.7 %)
Duration of gastric cancer (months)			
Mean^b^	8.82 (18.25)	6.89 (13.70)	7.87 (16.17)
Median^c^	0.69 (0–124.3)	0.62 (0–64.4)	0.68 (0–124.3)
KPS			
≥80	115 (96.6 %)	108 (93.9 %)	223 (95.3 %)
<80	4 (3.4 %)	7 (6.1 %)	11 (4.7 %)
Weight loss in prior 3 months			
≤5 %	70 (58.8 %)	65 (56.5 %)	135 (57.7 %)
5–10 %	28 (23.5 %)	28 (24.3 %)	56 (23.9 %)
>10 %	21 (17.6 %)	22 (19.1 %)	43 (18.4 %)
Primary tumor site			
Gastroesophageal junction	20 (16.8 %)	29 (25.2 %)	49 (20.9 %)
Fundus	12 (10.1 %)	9 (7.8 %)	21 (9.0 %)
Antrum	44 (37.0 %)	41 (35.7 %)	85 (36.3 %)
Body	43 (36.1 %)	37 (32.2 %)	80 (34.2 %)
Other	13 (10.9 %)	6 (5.2 %)	19 (8.1 %)
Unknown	1 (0.8 %)	2 (1.7 %)	3 (1.3 %)
Disease status			
Recurrent	30 (25.2 %)	26 (22.6 %)	56 (23.9 %)
Metastatic	89 (74.8 %)	89 (77.4 %)	178 (76.1 %)
Histology			
Adenocarcinoma			
Moderate differentiation	19 (16.0 %)	18 (15.7 %)	37 (15.8 %)
Moderate–low differentiation	6 (5.0 %)	8 (7.0 %)	14 (6.0 %)
Low differentiation	60 (50.4 %)	55 (47.8 %)	115 (49.1 %)
Unknown differentiation	25 (21.0 %)	27 (23.5 %)	52 (22.2 %)
Signet ring cell cancer	20 (16.8 %)	15 (13.0 %)	35 (15.0 %)
Mucous adenocarcinoma	3 (2.5 %)	4 (3.5 %)	7 (3.0 %)
Other	1 (0.8 %)	1 (0.9 %)	2 (0.9 %)
AJCC staging			
III	5 (4.2 %)	2 (1.7 %)	7 (3.0 %)
IV	113 (95.0 %)	112 (97.4 %)	225 (96.2 %)
Unknown	0	1 (0.9 %)	1 (0.4 %)
No. of organs involved			
1	52 (43.7 %)	56 (48.7 %)	108 (46.2 %)
2	37 (31.1 %)	42 (36.5 %)	79 (33.8 %)
>2	28 (23.5 %)	16 (13.9 %)	44 (18.8 %)
Prior therapy			
Radiotherapy	1 (0.8 %)	0	1 (0.4 %)
Surgery	46 (38.7 %)	39 (33.9 %)	85 (36.3 %)
Palliative	9 (19.6 %)	10 (25.6 %)	19 (22.4 %)
Curative	30 (65.2 %)	27 (69.2 %)	57 (67.1 %)
Other	7 (15.2 %)	2 (5.1 %)	9 (10.6 %)
Chemotherapy^d^	23 (19.3 %)	22 (19.2 %)	45 (19.2 %)

*AJCC* American Joint Committee on Cancer, *CF* cisplatin and 5-fluorouracil, *DCF* docetaxel, cisplatin, and 5-fluorouracil, *HR* hazard ratio, *KPS* Karnofsky performance status

^a^The range (years) is given in *parentheses*.

^b^The standard deviation is given in *parentheses*.

^c^The range (months) is given in *parentheses*.

^d^Adjuvant/neoadjuvant

### Treatment characteristics

Overall, 591 cycles of mDCF regimen treatment (median five cycles; range 1–13 cycles) and 464 cycles of CF treatment (median four cycles; range 1–12 cycles) were administered. The median duration of therapy was 17 weeks with the mDCF regimen (range 3–46 weeks) and 12.6 weeks with CF (range 3–40 weeks). The median actual and relative dose intensities of docetaxel were 288.7 mg/m^2^ (range 58–769 mg/m^2^) and 91.0 mg/m^2^ (range 61–104 mg/m^2^), respectively. The median actual and relative dose intensities of 5-FU and cisplatin were similar in both arms. Overall, 79.0 and 68.7 % of the patients in the mDCF regimen arm and the CF arm, respectively, had a cycle delay or a dose reduction (Table S1). Myelosuppression was the commonest reason for cycle delay in both treatment arms and the main reason for docetaxel dose reduction (2.9 %). None of these patients in either arm received concomitant radiotherapy.

At the cutoff date (the date when the last patient completed the study treatment), all patients had stopped the study treatment and 31.09 and 41.74 % of the patients in the mDCF regimen arm and the CF arm, respectively, discontinued treatment because of disease progression, whereas the remaining patients in both arms discontinued treatment for other reasons. As of the study cutoff date, 37.0 and 39.1 % of the patients in the mDCF regimen arm and the CF arm, respectively, received further antitumor therapy. Six patients (5.0 %) and five patients (4.3 %) in the mDCF regimen arm and the CF arm, respectively, underwent tumor-related surgery. Additionally, five patients (4.2 %) and nine patients (7.8 %) in the mDCF regimen arm and the CF arm, respectively, received tumor-related radiotherapy. Moreover, 31.9 and 35.7 % of the patients in the mDCF regimen arm and the CF arm, respectively, received tumor-related drug therapy after the end of the study treatment.

### PFS and OS

**Fig. 2:**
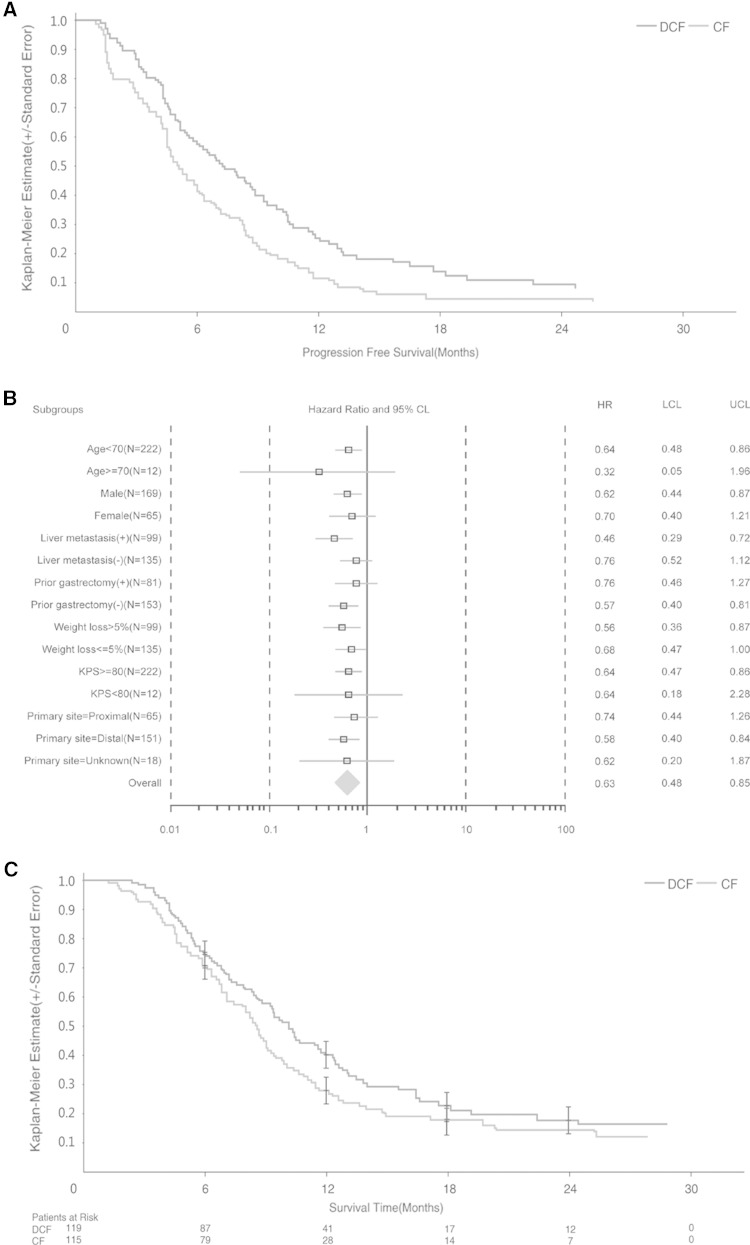
**a** The Kaplan–Meier distribution of progression-free survival (PFS). Patients with advanced gastric adenocarcinoma or adenocarcinoma of the gastroesophageal junction were randomly assigned to receive docetaxel, cisplatin, and 5-fluorouracil (*DCF*) or cisplatin and 5-fluorouracil (*CF*). **b** PFS [hazard ratios (*HR*) and 95 % confidence intervals (*CI*)] for selected subgroup analyses. **c** The Kaplan–Meier distribution of overall survival (OS). *KPS* Karnofsky performance status, *LCL* lower confidence limit, *UCL* upper confidence limit

The median duration of the follow-up was 22.5 months in the mDCF regimen arm and 23.5 months in the CF arm. The Kaplan–Meier distribution of PFS in the intention-to-treat population is shown in Fig. 2a. The median PFS was 7.2 months [95 % confidence interval (CI) 5.5–8.8 months] in the mDCF regimen arm and 4.9 months (95 % CI 4.5–6.0 months) in the CF arm. The difference in PFS between the two study arms was statistically significant (log-rank test, *P* = 0.0008) with a hazard ratio (HR) of 0.58 (95 % CI 0.42–0.80), and a risk reduction of 42 %. Subgroup analyses revealed that PFS was significantly longer for the following groups: age below 70 years (HR 0.64; 95 % CI 0.48–0.86), male gender (HR 0.62; 95 % CI 0.44–0.87), liver metastasis (HR 0.46; 95 % CI 0.29–0.72), no prior gastric surgery (HR 0.57; 95 % CI 0.40–0.81), weight loss more than 5 % (HR 0.56; 95 % CI 0.36–0.87), KPS ≥ 80 (HR 0.64; 95 % CI 0.47–0.86), and distal primary tumor site (HR 0.58; 95 % CI 0.40–0.84) (Fig. 2b).

The Kaplan–Meier distribution of OS is shown in Fig. 2c. The median OS was 10.2 months (95 % CI 8.6–11.9 months) in the mDCF regimen arm and 8.5 months (95 % CI 7.1–9.5 months) in the CF arm. The difference between the two arms was statistically significant (log-rank test, *P* = 0.0319), with an HR of 0.71 (95 % CI 0.52, 0.97) and a risk reduction of 29 %.

### ORR and TTF

**Fig. 3:**
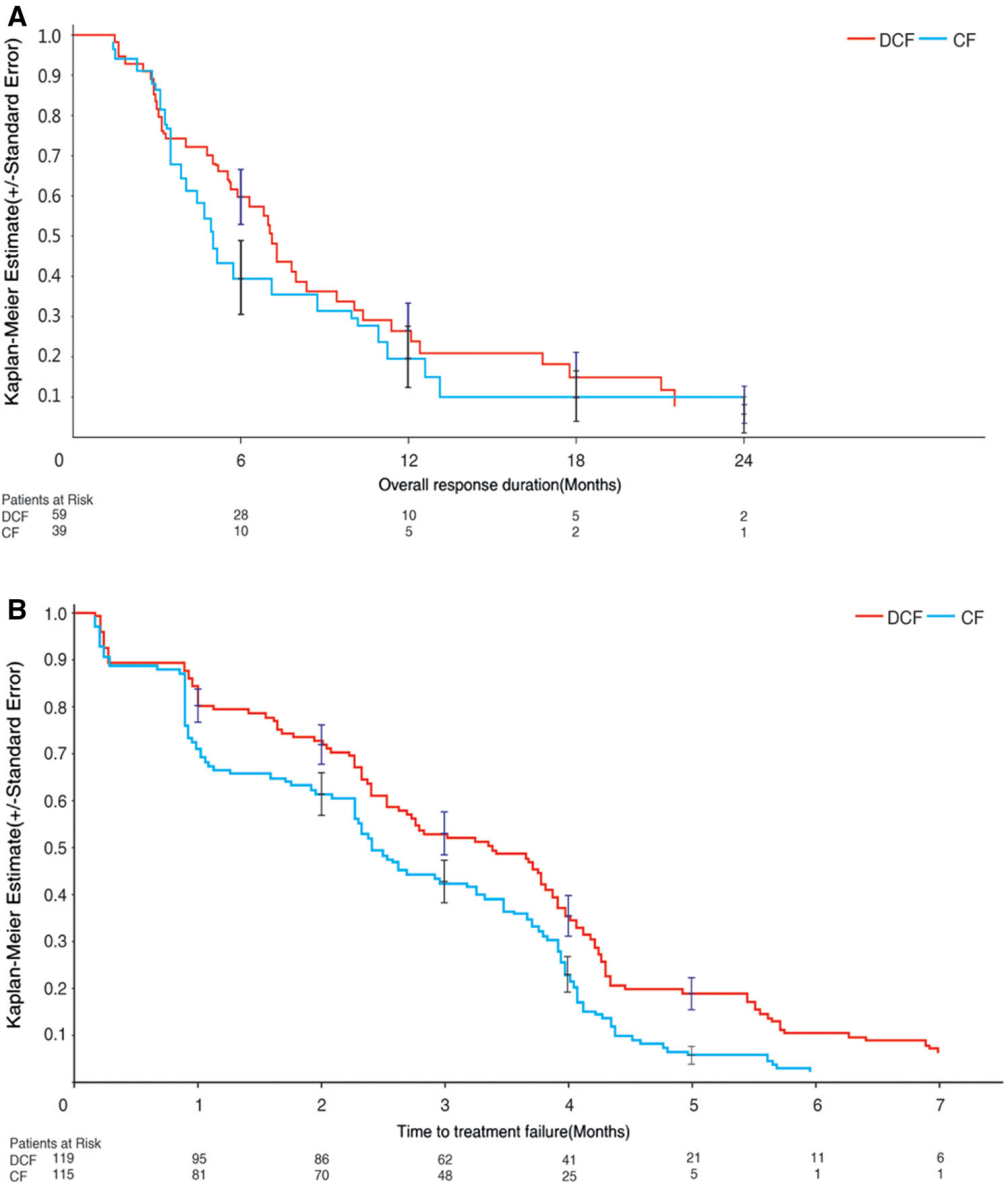
**a** The Kaplan–Meier distribution of overall response duration. Patients with advanced gastric adenocarcinoma or adenocarcinoma of the gastroesophageal junction were randomly assigned to receive docetaxel, cisplatin, and 5-fluorouracil (*DCF*) or cisplatin and 5-fluorouracil (*CF*). Duration of response was calculated in responders and was defined from the onset of partial response/complete response. **b** The Kaplan–Meier distribution of the time to treatment failure

The ORR (CR and PR) was 48.7 % (95 % CI 39.5–58.1) in the mDCF regimen arm, which was statistically significantly higher than that of the CF arm [33.9 % (95 % CI 25.3–43.3)] (Fisher’s exact test, *P* = 0.0244). The median overall response duration was 7.1 months (95 % CI 5.5–9.4 months) in the mDCF regimen arm and 5.0 months (95 % CI 3.5–8.7 months) in the CF arm (Fig. 3a). The difference between the two arms was not statistically significant (log-rank test, *P* = 0.3757), with an HR of 0.801 (95 % CI 0.489–1.312) and a risk reduction of 19.9 %.

The median TTF was 3.4 months (95 % CI 2.5–3.8 months) in the mDCF regimen arm and 2.4 months (95 % CI 2.3–3.2 months) in the CF arm (log-rank test, *P* = 0.0027), with an HR of 0.674 (95 % CI 0.518–0.876) and a risk reduction of 32.6 % (Fig. 3b), suggesting a statistically significant benefit in favor of the mDCF regimen.

### Safety

The main medication-related treatment-emergent hematologic and gastrointestinal adverse events are summarized in Table 2. Grade 3 and grade 4 neutropenia was significantly more frequent in patients in the mDCF regimen arm (60.5 %) than in patients in the CF arm (9.6 %), as were febrile neutropenia (12.6 % in mDCF regimen arm vs 0 % in CF arm) and neutropenic infection (1.8 % in mDCF regimen arm vs 0 % in CF arm). Grade 3 or grade 4 treatment-emergent serious adverse events related to docetaxel were recorded in only 2.5 % of the patients. The number of discontinuations due to a treatment-emergent adverse event was similar in both arms. Four deaths (3.4 %) occurred within 30 days of the last infusion in the mDCF regimen arm and one death (0.9 %) occurred within 30 days of the last infusion in the CF arm. The number of deaths occurring beyond 30 days of the last infusion was 85 (71.4 %) in the mDCF regimen arm and 90 (78.3 %) in the CF arm. The main cause of death was disease progression in both arms (70.6 % in the mDCF regimen arm vs 76.5 % in the CF arm).

**Table 2:** Hematologic and gastrointestinal toxicities (National Cancer Institute of Canada Common Toxicity Criteria version 1.0)

Toxicity	DCF (*n* = 119)		CF (*n* = 115)	
	Grade 3–4	All grades	Grade 3–4	All grades
Hematologic toxicities^a^				
Neutropenia	72 (60.5 %)	94 (79.0 %)	11 (9.6 %)	67 (58.3 %)
Leucopenia	62 (52.1 %)	105 (88.2 %)	2 (1.7 %)	74 (64.3 %)
Anemia	6 (5.0 %)	40 (33.6 %)	6 (5.2 %)	36 (31.3 %)
Thrombocytopenia	2 (1.7 %)	26 (21.8 %)	5 (4.3 %)	37 (32.2 %)
Febrile neutropenia	15 (12.6 %)	16 (13.4 %)	0	1 (0.9 %)
Gastrointestinal toxicities				
Stomatitis	4 (3.4 %)	26 (21.8 %)	0	5 (4.3 %)
Diarrhea	15 (12.6 %)	57 (47.9 %)	0	9 (7.8 %)
Nausea	3 (2.5 %)	71.4 (85 %)	8 (7.0 %)	89 (77.4 %)
Vomiting	9 (7.6 %)	63 (52.9 %)	13 (11.3 %)	79 (68.7 %)

*CF* cisplatin and 5-fluorouracil, *DCF* docetaxel, cisplatin, and 5-fluorouracil

^a^Patients were assessed for hematologic toxicity if they had one or more cycles with a blood count for the given test between day 2 and the first infusion of the next cycle, and had received no prophylactic treatment during the cycle.

## Discussion

Docetaxel as a single agent has proven effective as first-line treatment in advanced gastric cancer in phase II clinical trials [22, 23]. The phase III V325 study has further demonstrated the clinical benefit of docetaxel [5]. The current study was conducted in previously untreated Chinese patients with advanced gastric adenocarcinoma using reduced doses of DCF. Consistent with previous findings [22], the mDCF regimen significantly improved PFS, OS, and RR, suggesting that the inclusion of docetaxel in the CF regimen is clinically beneficial to Chinese patients with advanced gastric cancer.

The median PFS was 2.3 months longer in the mDCF regimen arm compared with the CF arm, exceeding the preset superiority threshold of 1.9 months for the difference between the study arms and thus meeting the primary end point. Additionally, the mDCF regimen significantly prolonged the median OS compared with the CF regimen [10.2 months vs 8.5 months, HR 0.71 (95 % CI 0.52–0.97), *P* = 0.0319]. In terms of the reduction in the risk of death, our results were similar to those of the V325 study [5]. The V325 study enrolled 457 patients with advanced gastric cancer in 16 countries and randomized them to receive either docetaxel at 75 mg/m^2^ and cisplatin at 75 mg/m^2^ plus 5-FU at 750 mg/m^2^/day (DCF) or cisplatin at 100 mg/m^2^ plus 5-FU at 1,000 mg/m^2^/day and showed the efficacy of the DCF combination in patients with advanced gastric cancer [5]. After a median follow-up of 23.4 months, the median OS was 9.2 months in the DCF arm and 8.6 months in the control arm (log-rank test, *P* < 0.02), with a reduction of 23 % in the risk of death [5]. In our study, the patients assigned to receive the mDCF regimen achieved longer OS than the patients enrolled in the V325 study. Additionally, the ORR in both study arms was higher than in the V325 study (48.7 % in the mDCF regimen arm vs 30.3 % in the DCF arm; 33.9 % vs. 19.9 % in the CF arm). These differences may be explained by the higher number of patients with locally advanced cancer enrolled in our study (23.9 % vs. 3 % in the V325 study). Additionally the radical surgery, radical radiotherapy, or second-line chemotherapy recommended after chemotherapy in 30 % of patients in our study may have contributed to the prolongation of the OS. Although the OS of 10.2 months was longer than that in the V325 study, it was shorter than that observed in other studies in which patients with gastric cancer were treated with doublets [24–26]. The SPIRITS trial, in which 298 Japanese patients with advanced gastric cancer were randomized to receive either S-1 (Taiho Pharmaceutical, Tokyo, Japan) plus cisplatin or S-1 alone, showed a median OS of 13 months in the S-1 plus cisplatin arm and 11 months in the S-1 alone arm (*P* = 0.04) [24]. In the START trial, which enrolled 639 patients from Japan and Korea, the OS was 12.5 months in patients who received S-1 in combination with docetaxel compared with 10.8 months in patients who received S-1 alone [25]. In another phase III trial, published by Boku et al. [26], the median OS was 10.8 months in patients assigned to 5-FU treatment, 12.3 months in those assigned to irinotecan plus cisplatin treatment, and 11.4 months in those assigned to treatment with S-1 alone. The shorter OS in our study as compared with the OS in previous ones described here may be explained by the differences in the frequency of administration of the second-line treatment and which might have favorably influenced the OS in these studies: 35 % (chemotherapy, radiotherapy, and surgery) in our study compared with up to 78 % of the patients assigned to doublets in other studies [24–26]. Thus, although the second-line therapy may have contributed to the prolongation of the OS in our study, the low number of patients receiving second-line therapy was due to the study starting in 2008 when the proportion of second-line treatment acceptance was generally low in China and limited the effect.

It should be noted that we chose the PFS as the main objective of our trial instead of the OS owing to the requirement of the China Food and Drug Administration to have this parameter evaluated as the main objective in clinical trials performed in China before a drug or a regimen obtains marketing approval. The MAGIC study [27] proved that perioperative chemotherapy with a regimen of epirubicine, cisplatin, and continuous 5-FU infusion enhanced the R0 rate (no residual tumor) and prolonged OS. In our study, the mDCF regimen achieved a tumor RR of nearly 50 %. Although our sample size was limited and we did not analyze locally advanced cancer patients who received radical surgery after chemotherapy, on the basis of the higher tumor RR, it is worth investigating further whether the mDCF regimen could be used as neoadjuvant chemotherapy.

The clinical benefit of DCF in the V325 study was obtained at the expense of increased toxicity [5], which requires comprehensive toxicity management strategies. As expected, in Chinese advanced gastric cancer patients, the mDCF regimen did not cause any previously unreported treatment-emergent adverse events. Compared with CF, specific adverse events such as leukopenia and diarrhea were more frequent with the mDCF regimen, but other adverse events such as nausea and dyspepsia were less frequent. This toxicity profile in Chinese advanced gastric cancer patients treated with the mDCF regimen was similar to that of the V325 study.

Predictably, for docetaxel-containing regimens, the most frequently reported toxicities are hematologic ones. In the V325 study, the frequency of grade 3/4 neutropenia was 82 % and that of febrile neutropenia was 29 % in the DCF arm, whereas in our study, for the mDCF regimen arm, the frequencies of both grade 3/4 neutropenia and febrile neutropenia were lower (60.5 and 13.4 %, respectively), indicating that the mDCF regimen was associated with reduced hematologic toxicities as compared with DCF in the V325 study without compromising efficacy.

Although we have shown good efficacy and acceptable toxicities with the mDCF regimen, we must acknowledge several limitations resulting from the open-label design of our study. Tumor response and PFS were evaluated by investigators, and although much effort has been done to limit the bias in the evaluation of these parameters (standardized criteria were used to assess the response and disease progression; tumor evaluation was performed at prespecified time points), the interpretation of the imaging findings may have differed between investigators. Although the blinding of clinical trials is the recommended procedure to prevent systematic bias [28], clinical trials for which the investigational medical product is administered intravenously are often designed as open-label trials, especially in oncology. In our study, the docetaxel added to cisplatin and 5-FU in the mDCF regimen arm was administered as an intravenous infusion, and this would have made the blinding of the study treatment difficult.

In conclusion, our study demonstrated the superior efficacy in terms of PFS of the mDCF regimen versus CF as first-line treatment for advanced gastric cancer. The study results document the value of docetaxel in the treatment of Chinese advanced gastric cancer patients, and the mDCF regimen is comparable in efficacy to DCF used in the V325 study but has a more favorable safety profile.

## Electronic supplementary material

Below is the link to the electronic supplementary material.

Supplementary material 1 (DOC 28 kb)

Supplementary material 2 (DOC 56 kb)
