# Supplementary material for: Randomized multicenter phase III study of a modified docetaxel and cisplatin plus fluorouracil regimen compared with cisplatin and fluorouracil as first-line therapy for advanced or locally recurrent gastric cancer
Source: Gastric Cancer. 2015 Jan 21;19(1):234–44. doi: 10.1007/s10120-015-0457-4 (PMC4688303; doi:10.1007/s10120-015-0457-4)
Supplement: Supplementary file 1 — Supplementary material 1 (DOC 28 kb) [file 10120_2015_457_MOESM1_ESM.doc]

**Supplementary Material**

***Inclusion and exclusion criteria***

A patient was eligible for the study 1) if the patient was at least 18 years of age and had pathologically proven gastric adenocarcinoma, including adenocarcinoma of the gastroesophageal junction; 2) if the patient had at least one measurable lesion according to the RECIST; 3) if the patient had a Karnofsky performance status (KPS) score ≥70; 4) if the patient had a life expectancy over 3 months; 5) if the patient had adequate hematologic parameters and hepatic and renal function; 9) if the patient did not receive prior palliative therapy, but previous adjuvant (neo-adjuvant) chemotherapy was allowed if more than 6 months had elapsed between the end of the adjuvant (neoadjuvant) therapy and the first relapse.

A patient was excluded from the study 1) if the patient was pregnant, lactating, or fertile but did not take adequate contraceptive measures; 2) if the patient had received prior treatment with taxanes or CDDP as adjuvant (and/or neoadjuvant) chemotherapy with a cumulative dose >300 mg/m^2^, or if the patient had ≥2 prior adjuvant or neoadjuvant treatment regimens; 4) if the patient had known brain or leptomeningeal metastasis or at least grade 2 symptomatic peripheral neuropathy according to the National Cancer Institute of Canada Common Toxicity Criteria (NCIC-CTC).
