# Supplementary material for: Randomized multicenter phase III study of a modified docetaxel and cisplatin plus fluorouracil regimen compared with cisplatin and fluorouracil as first-line therapy for advanced or locally recurrent gastric cancer
Source: Gastric Cancer. 2015 Jan 21;19(1):234–44. doi: 10.1007/s10120-015-0457-4 (PMC4688303; doi:10.1007/s10120-015-0457-4)
Supplement: Supplementary file 2 — Supplementary material 2 (DOC 56 kb) [file 10120_2015_457_MOESM2_ESM.doc]

**Supplementary Table 1.** Cycles delayed and dose reductions

|  | *DCF (n=119)* | *CF (n=115)* |
| --- | --- | --- |
| **No. of total cycles received (%)**  No delay or dose reduction  Delay only  Dose reduction only  Both delay and dose reduction | 591(100%)  305(51.6%)  241(40.8%)  15(2.5%)  30(5.1%) | 464(100%)  257(55.4%)  197(42.5%)  4(0.9%)  6(1.3%) |
| **No. of patients who received study chemotherapy**  At least 1 cycle delay  No cycle delay or dose reduction  Cycle delay only  Dose reduction only  Both delay and dose reduction | 119(100%)  90(75.6%)  25(21.0%)  63(52.9%)  4(3.4%)  27(22.7%) | 115(100%)  78(67.8%)  36(31.3%)  72(62.6%)  1(0.9%)  6(5.2%) |

CF, cisplatin and fluorouracil; DCF, docetaxel, cisplatin, and fluorouracil

**Supplementary** **Table 2.** Patient and hazard ratio data for each clinical benefit endpoint

| *Variable* | *DCF (n=119)* | *CF (n=115)* |
| --- | --- | --- |
| Definite worsening of KPS^ [n(%)] | 27(22.7) | 28(24.3) |
| Deterioration | 24(20.2) | 25(21.7) |
| Death | 3(2.5) | 3(2.6) |
| Censored | 92(77.3) | 87(75.7) |
| HR(95% CI) | 0.858(0.505, 1.457) |  |
| Relative risk reduction (%, 95% CI) | 14.2(-45.7,49.5) |  |
| Log rank test, *P* | 0.5698 |  |
| Definite worsening of weight loss* [n(%)] |  |  |
| Deterioration | 27(22.7) | 40(34.8) |
| Death | 25(21.0) | 36(31.3) |
| Censored | 92(77.3) | 75(65.2) |
| HR(95% CI) | 0.490(0.362,0.963) |  |
| Relative risk reduction (%, 95% CI) | 41.0(3.7, 63.8) |  |
| Log rank test, *P* | 0.0324 |  |
| Definite worsening of appetite^#^ [n(%)] | 35(21.0) | 34(29.6) |
| Deterioration | 20(16.8) | 30(26.1) |
| Death | 5(4.2) | 4(3.5) |
| Censored | 94(79.0) | 81(70.4) |
| HR(95% CI) | 0.654(0.390, 1.096) |  |
| Relative risk reduction (%, 95% CI) | 34.6(-9.6,61.0) |  |
| Log rank test, *P* | 0.1035 |  |
|  | TCF (n=44) | CF (n=45) |
| Median pain free survival^&^ (months)(95% CI) | 7.0(5.3, 11.4) | 8.3(6.2, 10.5) |
| HR(95% CI) | 1.015(0.634, 1.623) |  |
| Relative risk reduction (%, 95% CI) | -1.5(46.6, -62.3) |  |
| Log rank test, *P* | 0.9509 |  |

CF, cisplatin and fluorouracil; DCF, docetaxel, cisplatin, and fluorouracil; HR, hazard ratio; KPS, Karnofsky performance status.

^Worsening was defined as a definitive decrease in performance status by 1 KPS category compared with baseline.

*Definite weight loss was defined as a decrease in weight by at least 5% compared with baseline.

^#^Definitive worsening of appetite was defined as a decrease of appetite by ≧1 grade compared with baseline.

^&^ Pain-free survival was defined as the interval between random assignment and the appearance of grade ≧1 cancer pain and was assessed only in patients with grade 0 cancer pain at baseline.
